# Effective connectivity analysis explains metastable states of ongoing activity in cortically embedded systems of coupled synfire chains

**DOI:** 10.1186/1471-2202-16-S1-P61

**Published:** 2015-12-18

**Authors:** Chris Trengove, Cees van Leeuwen, Markus Diesmann

**Affiliations:** 1 Perceptual Dynamics Laboratory, University of Leuven, Leuven, Flemish Brabant, B3000, Belgium; 2 Institute of Neuroscience and Medicine (INM-6) and Institute for Advanced Simulation (IAS-6), Jülich Research Centre and JARA, Jülich, Germany; 3 Department of Psychiatry, Psychotherapy and Psychosomatics, Medical Faculty, RWTH Aachen University, Aachen, Germany; 4 Department of Physics, Faculty 1, RWTH Aachen University, Aachen, Germany

## 

In models of the cortex, synaptic connectivity is often assumed to be random within broad constraints such as interlayer connection densities. Alternatively, the connectivity could include richly structured circuitry. A recent study [1] demonstrated this in a model of local cortex of order 1 mm^3 ^in size: a large number of synfire chains (small pools of neurons sequentially linked by feedforward connections) activated by waves (sequences of propagating spike packets) were embedded in a recurrent network of excitatory and inhibitory neurons. The model exhibits stable global dynamics in the asynchronous irregular regime and stable propagation of multiple synfire waves. Background noise generated by waves destabilizes wave propagation, providing a negative feedback signal limiting their number.

Here we add inter-chain couplings (each chain branches to two successors) and variability in chain strengths to obtain a recurrent system with a topography. We study emergent patterns of activity propagation in such a system. Ongoing endogenous activity due to branching of waves and regulation of wave activity by noise feedback is typically found. Across model realizations and runs, ongoing activity manifests diverse steady-state patterns and transitions. We argue that steady states arise jointly and consistently with an *effective *connectivity: strength-dependent chain traversal probabilities averaged over noise fluctuations. By excluding chains with sub-threshold traversal probability we derive a family of effective coupling graphs (ECGs) parameterized by the global activity level. The distribution of wave activity in steady states is largely confined to the islands of circulation: strongly connected components (SCCs) and their associated out-components (OCs) in optimally chosen ECGs. A condensed ECG (cECG) allows the relationship between activity and effective connectivity to be visualized (Figure 1).

**Figure 1:**
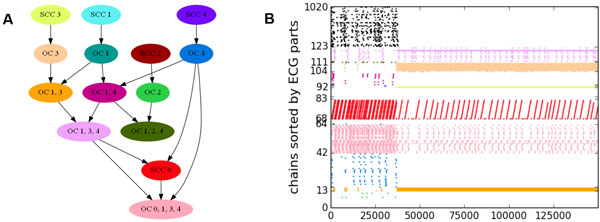
**A The cECG with SCCs and OCs partitioned by their overlaps as nodes**. **B **Chain indices of traversal events versus time (ms), permuted so chains in each cECG node are adjacent and colored accordingly.

The system is implemented both as a large-scale network of integrate-and-fire neurons and as a reduced model with binary-state pools as basic units. The reduced model exhibits activity patterns very similar to those of the full model and provides a valuable tool for studying the latter. We propose that the principle whereby activity patterns arise in concert with dynamically tuned effective connectivity applies to a broad class of networks with complex topologies.

## References

[B1] Trengove C van Leeuwen C Diesmann M High capacity embedding of synfire chains in a cortical network model J Comput Neurosci 2013 34 2 185 209 22878688 10.1007/s10827-012-0413-9 PMC3605496

